# Preventing Disease

**Published:** 1861-05

**Authors:** 


**PREVENTING DISEASE.** — To inaugurate a healthy, moral condition of the community, we must prevent crime by educating the masses to its avoidance. This is the part of the true philanthropist; so is it the highest aim of the good physician, not to cure disease, but to prevent it. Such was the announcement in the very first line of the first number of our JOURNAL. On the title-page we intimated that the good time was coming, and we are gratified at some signs of its approach, infinitely more tangible than any Dr. Cummings can show that the world is near its end. In the great city of London, which now covers a space of one hundred and twenty-one square miles, on which live two and three quarter millions of people, one person out of every dozen dies in a workhouse, one out of every half-dozen in a charitable institution, one person out of every six is too poor to die in his own bed, and must rest in a pauper's grave. To what extent sickness and slow disease incapacitate the striving multitudes from supporting themselves, none can estimate better than the physician and the philanthropist. In view of so sad a result in the greatest city of the greatest nation on earth, the Registrar-General first looks for relief in improving the physical health of the teeming millions, and to this end aims at securing three things:

1. Pure air to breathe.
2. Pure water to drink.
3. A healthy soil to live upon.

The next thing he proposes is, to set the two thousand doctors of London to work in teaching the people how to prevent disease. And he wakes up to this as a "bran-new" idea. In the vividness of his fancy he fairly runs riot in beatific anticipations. "Imagine an army of two thousand of the most enlightened profession in the world employed in instructing the people in the way of a healthy life. How many thousands of lives would be saved every year in London alone! How much better and happier the population would be!" The majestic "Thunderer," the London *Times*, chimes in with the Registrar-General, and heads it, a "Beginning of the Movement under Sir B. Hall's Act." The *Times* has made a mistake. It is Dr. W. W. Hall, 42 Irving Place, New-York, and not an English baronet who set the ball in motion. Think a letter to her majesty Queen Vic the First might rectify matters and put the glory on the right cranium. The Registrar-General complains that: "Physicians are chiefly employed in treating disease. The art of preventing is not cultivated; it is not taught in any of our medical schools; it is not formally the subject of examination in any of our universities. The father of a family does not go to a doctor and say: 'How can I preserve my health and make my children well and vigorous, and develop all their faculties to the fullest extent?'" The *Times* goes on to say that: "Under Sir B. Hall's Act, medical health officers are appointed in the various districts of London, and many of them are working courageously against ignorant opposition with success. They deserve public approbation, for they have done quietly a great deal of good work, and it is probable have saved many lives and prevented much sickness."

The readers of this JOURNAL will bear witness to its steady efforts to teach them how to prevent disease; and we feel safe in saying that tens of thousands in this country have learned useful lessons in this direction from these pages. What is most deeply to be regretted is, that so few persons, comparatively, can be induced to pay a dollar a year in order to place in the hands of their children a publication devoted to the one point—how health may be maintained; how a good constitution can be preserved, counseling at the same time that when there is actual disease, a regularly educated physician should be promptly called in.

